# Partnership Context of First Births in Russia: The Enduring Significance of Marriage

**DOI:** 10.1007/s10680-021-09600-5

**Published:** 2021-12-10

**Authors:** Evgeny M. Andreev, Elena Churilova, Aiva Jasilioniene

**Affiliations:** 1 International Laboratory for Population and Health, National Research University Higher School of Economics, Moscow, Russia; 2 Max Planck Institute for Demographic Research, Rostock, Germany

**Keywords:** First births, Marriage, Premarital conceptions, First birth interval, Russia, Shotgun marriage

## Abstract

While cohabitation and non-marital childbearing have been increasing in Russia since 1990, the share of marital first births that are conceived prior to marriage has changed very little since the Soviet era. The prior findings on the stability of trends in premarital conceptions in Russia have been contradictory and inconclusive. This study aims to extend the existing empirical evidence on premarital conceptions in Russia and to contribute to the discussion on the persistence of marriage as the preferred partnership context for parenthood. We focus on births that occurred within the first two years of marriage, and compare the childbearing patterns of Russian women who married in different historical periods. For our investigation of fertility among marital cohorts who married during the Soviet era (1960–1991), we use individual-level data from the 1994 microcensus. For our examination of fertility among more recent marital cohorts (2000, 2011, and 2016), we draw on data from birth records in civil registers. We also use relevant complementary data sources. Our findings show that there has been a marked shift in the relationship between conception and marriage in Russia. Increasingly, conceptions have been occurring before marriage, and in the most recent marital cohorts, the level of premarital first conceptions has even surpassed the level of marital first conceptions. The average interval between conception and entry into marriage has also been lengthening. We describe this unique pattern of childbearing and discuss some potential explanations for the ongoing association between marriage and childbearing in Russia.

**Supplementary Information:**

The online version contains supplementary material available at 10.1007/s10680-021-09600-5.

## Introduction

Over the past three decades in Russia, family formation and fertility patterns have changed substantially. Cohabitation has become more widespread, non-marital births have increased, and people are increasingly postponing marriage and childbearing. As in other post-Soviet countries, these changes in Russia were greatly influenced by the breakup of the Soviet Union (USSR) and by the societal transformations that followed (Vishnevsky, 2006). In the period between the mid-1980s and the beginning of the 2000s, marriage and fertility in Russia underwent changes that were more profound than those that occurred in the preceding 50 years. The proportion of individuals who cohabit in their first partnership has been increasing steadily in Russia since the early 1980s and exceeded 50% by the end of the 1990s (Puur et al., 2012). At the same time, marriage risks among non-pregnant childless women have declined markedly since the mid-1990s (Hoem et al., 2009). The mean age at marriage as well as the mean age at childbearing has risen considerably, and childlessness has become increasingly widespread, especially in Moscow (Biryukova & Tyndik, 2015b).

The level of non-marital fertility started increasing in Russia in the mid-1980s, when it was around 11% (Zakharov & Churilova, 2013), and reached its highest point in 2005, at 30%. Although the share of non-marital births declined thereafter, it has remained relatively high and is currently well above 20% (Rosstat, 2019). Even so, the current level of non-marital childbearing in Russia is significantly lower than it is in most European countries (Eurostat, 2021), and, unlike in Western Europe, more than half of all non-marital births that occurred in Russia in 1968–2012 were to single mothers. Viewed from this perspective, the Russian non-marital fertility pattern in this period resembles that of the USA (Perelli-Harris & Gerber, 2011).

Given these changes in union formation and childbearing, combined with the increased access to modern contraception in Russia (Denisov et al., 2012; Vishnevsky et al., 2017), one could expect to observe that the proportion of premarital conceptions in the country has also declined. However, the share of premarital conceptions—which are often associated with unplanned pregnancies and which were relatively common in the Soviet period—has changed only slightly over time. According to the available research estimates, the share of first children who were conceived outside of marriage but were born within marriage was 46% in 1981, 48% in 1998 (Tolts et al., 2006), and 49% in 2011 (Churilova & Chumarina, 2014).

There is no solid explanation for the stability of the relatively large proportion of premarital conceptions in contemporary Russia. On the one hand, the connotations of a “shotgun marriage” have changed significantly since the Soviet era. Whereas most premarital pregnancies in the past were unplanned, today it is relatively common for such a pregnancy to be the outcome of a rational decision made by both partners (Chernova & Shpakovskaya, 2010). It has been shown that even among highly educated women, who are more likely than their less educated counterparts to use modern contraception, a significant share of the first births that occur within marriage results from premarital conceptions (Churilova & Chumarina, 2014). On the other hand, while there is some regional variation in family formation and reproductive behaviour, the value placed on legal marriage remains very high throughout Russian society. The high rates of marriage following a non-marital conception indicate that in Russia, traditional family formation patterns have endured (Birukova & Tyndik, ).

Research on premarital conceptions, as well as on non-marital conceptions in general, is still relatively scarce in Russia. Only a few studies have examined this topic explicitly (e.g. Biryukova & Tyndik, 2014, 2015a; Churilova & Chumarina, 2014; Tolts et al., 2005, 2006), and there are no existing studies that have investigated premarital conceptions in the second half of the 2010s. This study aims to extend the existing limited knowledge on premarital conceptions in Russia and to contribute to the discussion on the persistence of marriage in Russia as the preferred partnership context for parenthood. We focus on births that occurred within the first two years of marriage and compare the childbearing patterns of women who married in different periods of Russian history. Our analyses are based on data from multiple sources. For our investigation of fertility in marriages contracted in the Soviet period (1960–1991), we use individual-level data from the 1994 microcensus, whereas for our examination of fertility among the most recent marital cohorts (2000, 2011, and 2016), we use data from birth records of the civil registers. In addition, for our analyses we draw upon the available official vital statistics, relevant data from the 2002 and the 2010 population censuses and the 2015 microcensus, as well as data from selected national sample surveys.

## Background

### Understanding the Context of Non-marital Childbearing

The mid-1960s marks the point in time when the major transformation in union formation and childbearing behaviour began in Europe. These changes have unfolded very differently across European countries. According to the narrative of the second demographic transition (SDT), changes in fertility and family formation occur in sequence, with one event triggering the next (Van de Kaa, 2002). Before the onset of the SDT, premarital pregnancy and “forced” marriage were seen as undesirable and to be avoided. However, with the growing popularity and social acceptance of cohabitation, attitudes loosened, and marriage was often postponed until the bride became pregnant (Van De Kaa, 1997, 2002). The downward trend in the proportion of premarital conceptions was accompanied by an increase in the proportion of births that occurred within cohabitation (Gibson-Davis & Rackin, 2014; Holland, 2013).

The prevalence of non-marital births is associated with the perception and the role of cohabitation in family formation, which vary significantly across countries, from cohabitation being rare or being seen as a prelude to marriage, to cohabitation becoming indistinguishable from marriage (Heuveline & Timberlake, 2004). From a socio-cultural point of view, a large number of shotgun marriages in a society indicate that the social norm that marriage is the only living arrangement suitable for bearing a child remains strong in that context. Indeed, most European countries did not grant to children of unmarried parents the same legal rights as to children whose parents were married until the 1980s (Perelli-Harris & Sanchez-Gassen, 2012). In modern societies, individual norms and values also appear to play a significant role in family formation. Some unmarried couples rush to marry immediately after finding out about a pregnancy, because they believe that a child must be born in a marital union (Ipatova & Tyndik, 2015). The pressure to legitimate the relationship in the event of a pregnancy may also come from the parents of the young couple, because many members of the older generation continue to adhere to conservative social norms. In other words, young people may marry because they are afraid of parental and social disapproval (Manning et al., 2011). Alternatively, cohabiting partners may choose to marry simply because the country’s legal framework and existing policies do not adequately protect unmarried parents (Le Goff, 2002).

Another explanatory framework that is often employed in non-marital childbearing research is the “pattern of disadvantage”. This concept was initially built on empirical evidence from the USA (see Perelli-Harris et al., 2010). Unlike the SDT framework, which posits that highly educated individuals holding more liberal attitudes and values are at the forefront of the changes in family formation behaviour, the pattern of disadvantage framework associates non-marital childbearing with social and economic vulnerability. From this perspective, non-marital childbearing is expected to be more common among single mothers and among less educated and low-income individuals (England et al., 2012; Perelli-Harris & Gerber, 2011; Perelli-Harris et al., 2010**)**.

Lappegård et al. (2018) found that some dimensions of the SDT theory can be helpful in explaining variation in non-marital childbearing across countries, whereas the pattern of disadvantage approach provides a better explanation for the inter-individual variation—and, to some degree, for the inter-regional differences—in patterns of non-marital childbearing within a country. With respect to Russia, Isupova (2015) argued that even though both the SDT theory and the pattern of disadvantage concept seem to be relevant in the Russian context, the Russian experience cannot be explained by these two theories alone. While some cohabiters indeed have socio-economic characteristics (e.g. lower level of education, lower earning power) that make them less attractive in the marriage market or hinder them from establishing a stable partnership, for other cohabiters, the choice between marriage and cohabitation is more complex, involving, among other considerations, levels of inter-personal trust and economic security within the couple (Isupova, 2015).

### Marriage and Non-marital Fertility in Russia

#### The Soviet Era

Starting in the 1940s, in the Russian Soviet Federative Socialist Republic (RSFSR),^1^ as well as in the USSR (also called the Soviet Union) as a whole, civil marriage was obligatory for couples who lived together. The Soviet ideology was overwhelmingly pronatalist, and marriage and family were at the centre of this ideology. In 1944, several laws were enacted that were aimed at regulating and controlling people’s private lives. From that point until the collapse of the USSR, marriage was the only legal arrangement that ensured spousal rights and duties. During this period, all healthy childless married women and childless men aged 18–54(59) years were obliged to pay an additional 6% tax if they were childless (Barkalov & Darsky, 1994). From 1944 to 1968, children born outside of marriage were considered illegitimate and could not be adopted by their father or be given their father’s name.

Undesirable forms of family behaviour, including cohabitation, divorce, and childlessness, were not only discouraged by the legal system; they were punished by the Komsomol^2^ and the Communist Party of the Soviet Union (CPSU) by restricting people’s career opportunities and by depriving them of certain benefits under the Soviet social welfare system (such as the right to receive an apartment and the like). Thus, unmarried men and women had many incentives to get married in the event of an unplanned pregnancy.

However, these strict measures aimed at stamping out cohabitation and non-marital births were not fully effective. By 1959, the share of out-of-wedlock births in Soviet Russia had risen to 14% (Avdeev & Monnier, 2000). After the enactment in 1969 of a new family code, which liberalised the divorce process and granted unmarried parents the right to register their non-marital children jointly (Avdeev & Monnier, 2000), 36% of children born out of wedlock were registered by both parents (Zakharov & Churilova, 2013). From the 1960s through the 1980s, Russia was among the European countries with the highest proportions of non-marital births (Avdeev & Monnier, 2000).

However, despite the liberalisation of the legal framework for marriage and divorce in the Soviet Union, marriage continued to be nearly universal in Russia until the collapse of the USSR. The lack of efficient family planning also helped to sustain high levels of marriage. Abortion was the main means of birth control in Russia into the 1990s (Denisov et al., 2012) and was used mainly by married women with children (Barkalov & Darsky, 1994). Modern methods of contraception were used by only 40% of Soviet married women (Belova & Darsky, 1972) and were not promoted among adolescents and childless women (Barkalov & Darsky, 1994). In the absence of modern and effective methods of contraception, accidental first conceptions happened relatively frequently among young people and usually ended in shotgun marriages (Jasilioniene, 2007; Philipov & Jasilioniene, 2008). Marriage and childbearing remained closely connected in Russia through the 1980s (Zakharov, 2013).

#### The Period After 1990

After the collapse of the USSR, the marriage and fertility patterns in Russia changed dramatically. The Soviet laws disappeared along with the USSR, and Russians were gradually gaining the freedom to choose their own lifestyles and value systems. The transition to a market economy led to the eventual disappearance of most of the advantages associated with marriage. Cohabitation and non-marital childbearing have become increasingly widespread in Russia since 1990. Growing numbers of young men and women are cohabiting in their first union (Puur et al., 2012). Nevertheless, in Russia, cohabitation is still seen as a prelude to—and not as an alternative to—marriage. Half of all cohabiting couples marry within five years of moving in together (Zakharov, 2008).

The level of non-marital childbearing in Russia reached its highest point (30%) in 2005, and started decreasing thereafter. The decrease was partly caused by a concurrent increase in fertility that resulted mainly from rising numbers of second and third births, which rarely happen outside of marriage (Frejka & Zakharov, 2013). The observation that about 50% to 60% of all non-marital births in recent years were registered by a joint statement of the parents suggests that more than half of all of these non-marital births were to cohabiting couples (Rosstat 2015b).

Public opinion in Russia seems to reflect these apparent contradictions in family formation behaviour. On the one hand, most adult Russians have positive attitudes towards cohabitation, with 43% saying that they find having children outside of legal marriage acceptable (Levada Center, 2012,3). On the other hand, the results of national surveys on family, fertility, and reproductive plans conducted in 2009, 2012, and 2017^4^ repeatedly found that two-thirds of women are in favour of marriage as a first union (Rosstat, 2009, 2012, 2017). Finally, it appears that marriage is increasingly seen as the best setting for having children, as the share of the Russian population supporting this view increased from 54% in 2002 to 63% in 2018 (Levada Center, 2018).

The preference for marriage is also reinforced by the absence of any legal protections for non-marital unions. The latest Russian Family Code, adopted in 1995, recognises legal marriages only. This means that the rights and responsibilities of cohabiting partners—including the right to a share of jointly held property in the case of separation, or the right to inheritance in the case of a partner’s death—are not legally defined.

When we look at contraceptive behaviour in Russia, we see that induced abortions started to decline in the 1990s and that this trend accelerated significantly from the mid-2000s onwards. The Russian Reproductive Health Survey (RRHS) conducted in 2011 showed that 80% of never-married women were using modern and effective contraception, while slightly less than 11% were using traditional methods, and about 10% were not using any contraception (Vishnevsky et al., 2017). Among married and cohabiting women, 57% and 56%, respectively, were using modern methods, 14% and 15% were using traditional methods, and 28.5% and 28.7% were not using any contraception (Vishnevsky et al., 2017). According to unpublished data from the RRHS, the share of pregnant women who said their pregnancy was planned was 71% among married women and 62% among cohabiting women^5^ (RRHS, 2011).

To sum up, due to the lack of modern and effective contraception, an unplanned pregnancy was a relatively frequent event in Soviet Russia. Since there were important legislative and social incentives both for getting married and for having a child within marriage, couples generally preferred to marry in such cases. In modern Russia, young men and women often opt for cohabitation as a first partnership. As modern methods of contraception are used by the majority of both single and partnered women, the risk of unplanned pregnancy has decreased considerably. Nevertheless, in Russia, marriage (both the first marriage and subsequent marriages) continues to be highly valued and to be seen as the best setting for having and raising children.

## Data and Methods

The main objectives of the empirical part of our study are to examine the distribution of first births by the time elapsed after marriage; to determine the share of premaritally conceived births; and to investigate the changes in this share over time. We look at the first births that occurred within two years of marriage among women who married while under age 35. We selected a two-year follow-up period based on the results of our preliminary analysis of randomly selected marital cohorts, which showed that 75–80% of marital first births occurred during the first two years after marriage.

Two different data sources were used for the core part of the analysis. First, to investigate childbearing among couples who married in the Soviet era, we employed (anonymous) individual-level data from the 1994 microcensus. Then, to track the first births of women of the 2000, 2011, and 2016 marital cohorts, we used retrospective information from official (anonymous) individual-level birth record data for three time periods: 2000–2002, 2011–2013, and 2016–2018.

The 1994 microcensus was conducted in all regions of Russia, excluding the Chechen Republic, and covered 5% sample of the total population.^6^ The microcensus data contain self-reported information on each woman’s year and month of birth, as well as on the month and year of each of her marriages, and on the month and year of each of her births. We restricted our analysis to marriages contracted in the 1960–1991 period, because the period before 1960 was characterised by a very different set of demographic and political conditions (Scherbov & Van Vianen, 2001). In the data, there were 1,618,193 women in total who married for the first time between 1960 and 1991. Of these women, 1,102,669 (68%) gave birth to their first child within two years of marrying. For the cases in which a woman had her first birth in a second marriage (2.8% of marital first births), we used the year and month of the second marriage. Although the microcensus provides complete information on first and second marriages only, this is not a critical limitation, as we found that among the married women under age 35, the share of those who indicated that they were in a third or higher order marriage was only 0.12%.

Turning to our second data source, we note that official data on birth records in civil registers have been collected and stored by the Russian Federal State Statistics Service (hereafter, the Rosstat) since 1998. Anonymous electronic copies of these birth records include information on the date of birth and the date of the mother’s current marriage. For the period from 2000 onwards, the data cover the whole territory of Russia, except for the Chechen Republic, for which data from 2000 to 2003 are missing. Between 1999 and 2010, filling in the birth order field in a birth record in a civil register was no longer compulsory in Russia. Nevertheless, most regions continued collecting this information. Moreover, data on the biological birth order continued to be reported in the medical birth certificate. In 2011, birth order reporting was again made compulsory. Importantly, the birth record data include information on the mother’s current marriage, but not on the order of the marriage. Therefore, we analysed all marriages together, irrespective of their order.

We used the birth record data in the same way as the 1994 microcensus data. For our investigation of women in the 2000 marital cohort, we used birth record data for 2000–2002, i.e. we looked at first births that occurred within two years of marriage. The electronic copies of the birth records for these years contain the mother’s month and year of birth, the month and year of the mother’s current marriage, the month and year of the child’s birth, and the child’s birth order. For these reasons, information on the birth order was available for only 58.2% of births. For the regions with missing birth order information, births were split according to the birth order distribution in the regions for which birth order information was available. This approach was applied to Russian birth data of the same years in the Human Fertility Database (HFD), and the results were satisfactory (see Andreev, 2016). We tabulated all births by the mother’s age and the interval between marriage and birth, and then distributed births with unknown birth order across these categories proportionally, splitting them between known first and second births and higher-order births.

For the 2011 marital cohort of women, we used data from the birth records for 2011–2013. The birth records for these years include the complete dates of the mother’s and the child’s birth. The total number of births was 714,691. The birth order was known for 89.3% of all births. To obtain complete data coverage by birth order, we distributed births with an unknown birth order across the same categories as those used for the 2000–2002 birth record data.

For women in the 2016 marital cohort, we used data from the birth records for 2016–2018. The 2016 records include complete dates of marriages. The total number of births was 453,724. The birth order was known for 98.6% of these births.

For our analysis, we needed data on each woman’s date of birth, the date of her first marriage, and the date of the birth of her first child. However, the 1994 microcensus data and the 2000–2002 official birth record data included information only on the months and years of these events. Therefore, in order to estimate a woman’s age at first marriage, her age at first birth (in completed years), and the length of the interval between the two events (in completed months), we randomly generated an “exact day” of the event by applying the uniform distribution. The large sample size allowed us to produce reasonably accurate results for this purpose.

The same analytical approach was applied to data from all of the selected data sources. We analysed the distribution of births that occurred within two years of marriage and split the duration time into months, from zero to 23. We classified children who were born in the first seven and a half months of marriage as having been conceived before the marriage.^7^ By contrast, we classified children who were born in the second half of the eighth and subsequent months of marriage as having been conceived within the marriage.

In addition, we examined the first childbirths of women in the 1960–1991 marital cohorts (the 1994 microcensus data) and the 2000, 2011, and 2016 marital cohorts (the birth record data), while taking into account the urban–rural divide.

The main analysis based on the micro-level data was complemented with an analysis of official vital statistics on births and marriages produced by the Rosstat. These data were used to estimate the total number of births conceived outside of marriage and to calculate the relative frequency of non-marital births among the women of the marital cohorts in our sample, including the annual number of live births by age; the marital status of the mother and the birth order for the 1989–2018 period; and the annual number of marriages by age for 2000, 2011, and 2016.

When considering the official statistics on marriages, it should be noted that the statistical tabulations of marriage records in the 1999–2010 period were replaced by brief tables. These concise reports provide data on the number of marriages by only four age groups of the groom and the bride (under 18, 18–24, 25–34, and 35 and older), and the total numbers of marriages and remarriages. Therefore, for comparative purposes, we used data for only one year from this period, namely for 2001. To ensure uniformity, we divided the women of all marriage cohorts into these four age groups.

Finally, to shed more light on the changes in the characteristics of cohabiting couples in Russia, we examined data from the 2002 and 2010 population censuses and the 2015 microcensus (Rosstat, 2002, 2010; Rosstat, 2015a). We also used the Rosstat microdata access system to obtain distributions of partnered women by age, marital status, and educational attainment.

## Results

### Changes in the First Birth Interval

**Fig. 1:**
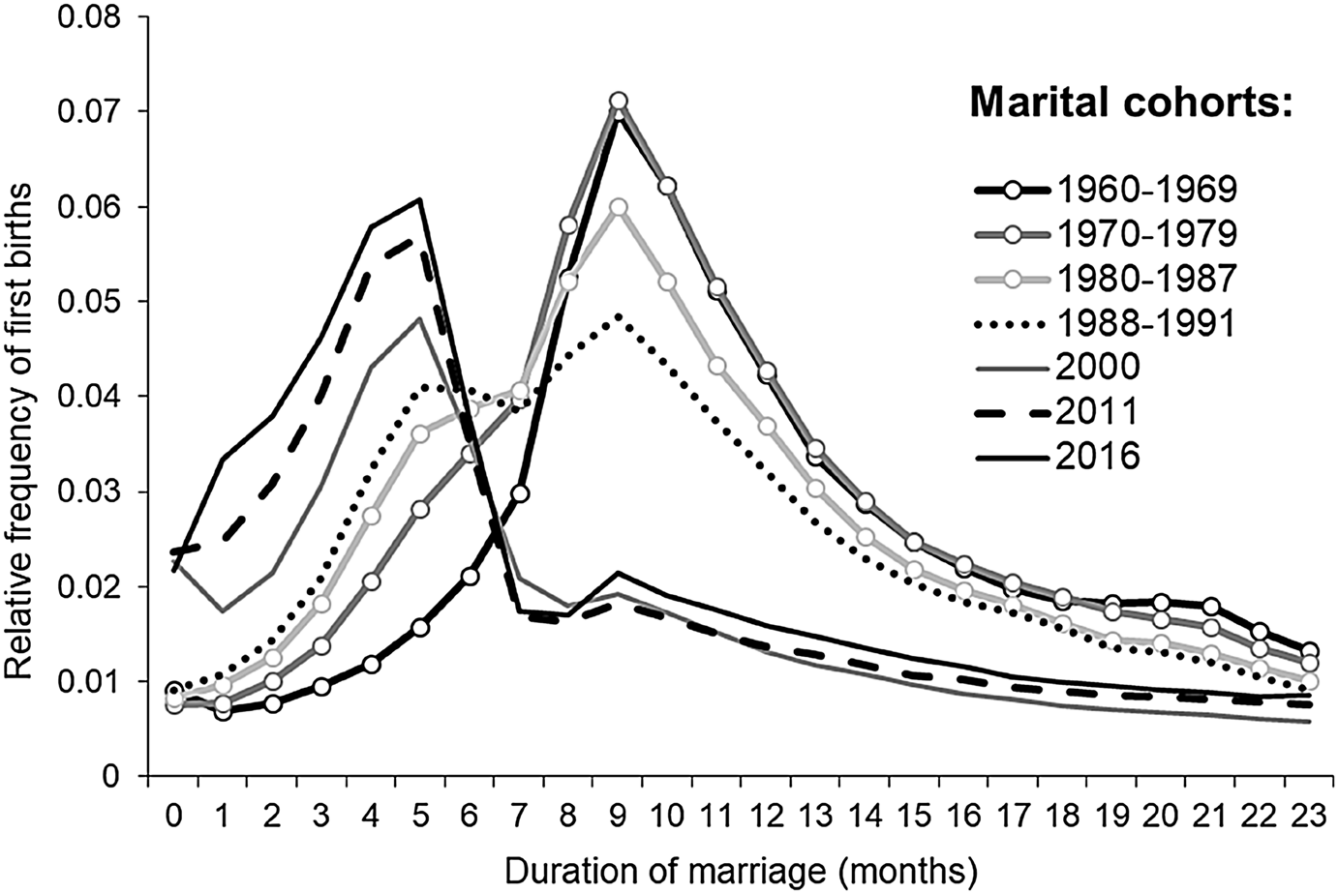
Relative frequency of first births born (within two years of marriage) to women who married at ages under age 35 by marital cohort and duration of marriage. *Data sources:* the 1994 microcensus; data on birth records for 2000–2002, 2011–2013, and 2016–2018; official statistics on the number of marriages by the age of the bride

Our examination of the frequency of births in relation to the duration of marriage revealed that significant changes occurred over the 1960–2016 period. Figure 1 shows the relative frequency of first births that occurred within two years of marriage to women (married at ages under age 35) of the selected marital cohorts. Among women who married in the 1960s, the largest number of first births occurred in the ninth month of marriage. More precisely, the peak was at 9.4 to 9.8 months of marriage. Among women of these marital cohorts, the likelihood that their first child would be born in the ninth month of marriage was 2.1–2.9 times higher than in any other month of the first two years of marriage. This trend was observed until about the 1987 marital cohort. Among the 1988–1991 marital cohorts, the distribution of first births became bimodal: i.e. the first child was most likely to be born in either the fifth or the ninth month of marriage. Among the 2000, 2011, and 2016 marital cohorts, the first child was most likely to be born in the fifth month. The distribution of first births regained a unimodal shape, with the peak shifting to the left, which indicated that, on average, the first child was born sooner after the marriage than was the case for the preceding marital cohorts of mothers.

**Fig. 2:**
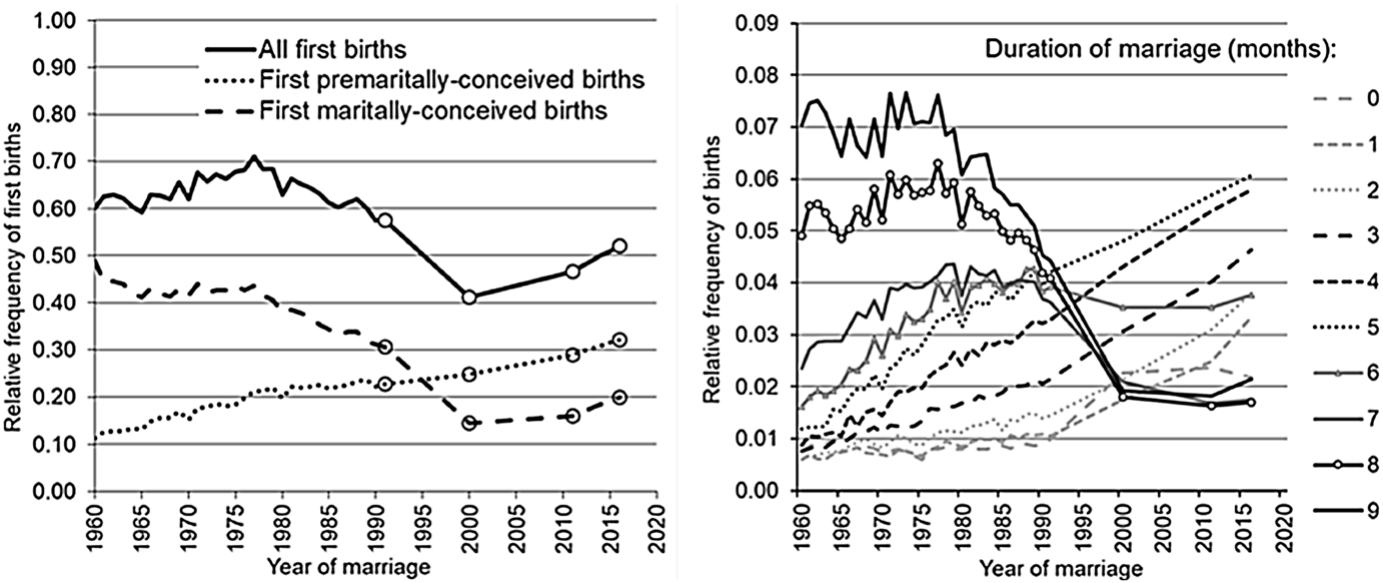
Relative frequency of first births born (within two years of marriage) to women who married at ages under age 35 by marital cohort and marital/non-marital conception (left panel) and by marital cohort and duration of marriage (right panel) *Data sources:* the 1994 microcensus; data on birth records for 2000–2002, 2011–2013, and 2016–2018; official statistics on the number of marriages by the age of the bride. *Note:* The time series are interrupted between 1991 and 2000, and again between 2000 and 2011

The changes in the pattern of the distribution of first births were brought about by several processes. The left panel of Fig. 2 presents the overall trend in the frequency of first births that occurred within two years of marriage and in the frequency of first births depending on whether the conception took place before or after the marriage. The overall trend in first births was shaped by the countervailing trends in first births conceived before and within marriage: over the marital cohorts covered in our analysis, the frequency of first births conceived before marriage increased 2.6-fold, whereas the frequency of first births conceived within marriage declined 2.7-fold (mainly among the marital cohorts after 1976). From the 1960 to the 2016 marital cohort, the average interval between the entry into marriage and the first birth decreased from 12.5 to 7.8 months (i.e. by 4.7 months). Over the same period, the share of children conceived within marriage among all first children born (within two years of marriage) to women who married while under age 35 declined sharply, from 81 to 39%.

One of the most striking findings is that the frequency of first births that occurred in the first five months of marriage was increasing steadily over our study period, with almost no interruptions (the right panel of Fig. 2). The frequency of first births that occurred in the sixth to seventh month of marriage—and, to some extent, also in the eight month of marriage—was increasing as well, but only until around the 1978 marital cohort; among subsequent cohorts, it decreased and stabilised at a level considerably below that observed among the 1960s marital cohorts. A similar pattern is apparent in the trends in first births that occurred between nine and 23 months of marriage, i.e. there was an accelerating decline that began roughly after the 1970s marital cohorts, with the level stabilising thereafter (see Appendix 1 in the online supplementary materials).

According to our estimates,^8^ the average length of pregnancy at the time of marriage was relatively stable in Soviet Russia, ranging from 3.2 to 3.4 months, and did not start to increase until around the mid-1980s. In the 1960 marital cohort, 11% of brides were, on average, 3.3 months pregnant at the time of marriage. In the 1988–1991 marital cohort, the proportion of brides who were pregnant increased to 26%, but the mean duration of the pregnancy increased only slightly, i.e. to 3.8 months. From the 1988–1991 to the 2000 marital cohort, the share of brides who were pregnant did not change, but the mean duration of the pregnancy at marriage increased to 4.5 months. During the 2000s, these trends did not change significantly. In the 2011 marital cohort, 29% of brides were pregnant, and the average length of the pregnancy at the time of marriage had increased to 4.7 months. In the 2016 marital cohort, the share of brides who were pregnant (30%) and the average length of pregnancy at the time of marriage (4.9 months) had not changed significantly.

The incidence of first births within two years of marriage was higher among women who married at ages 18–24 than among those who married at ages 25–34. However, the first birth frequency pattern in both age groups of women was relatively similar (see Appendix 2 in the online supplementary materials). Moreover, whereas the increase in the frequency of premarital conceptions was continuous across the marital cohorts among women who married before age 25, this increase did not become evident until the 1990s marital cohorts among women who married at ages 25–34.

**Fig. 3:**
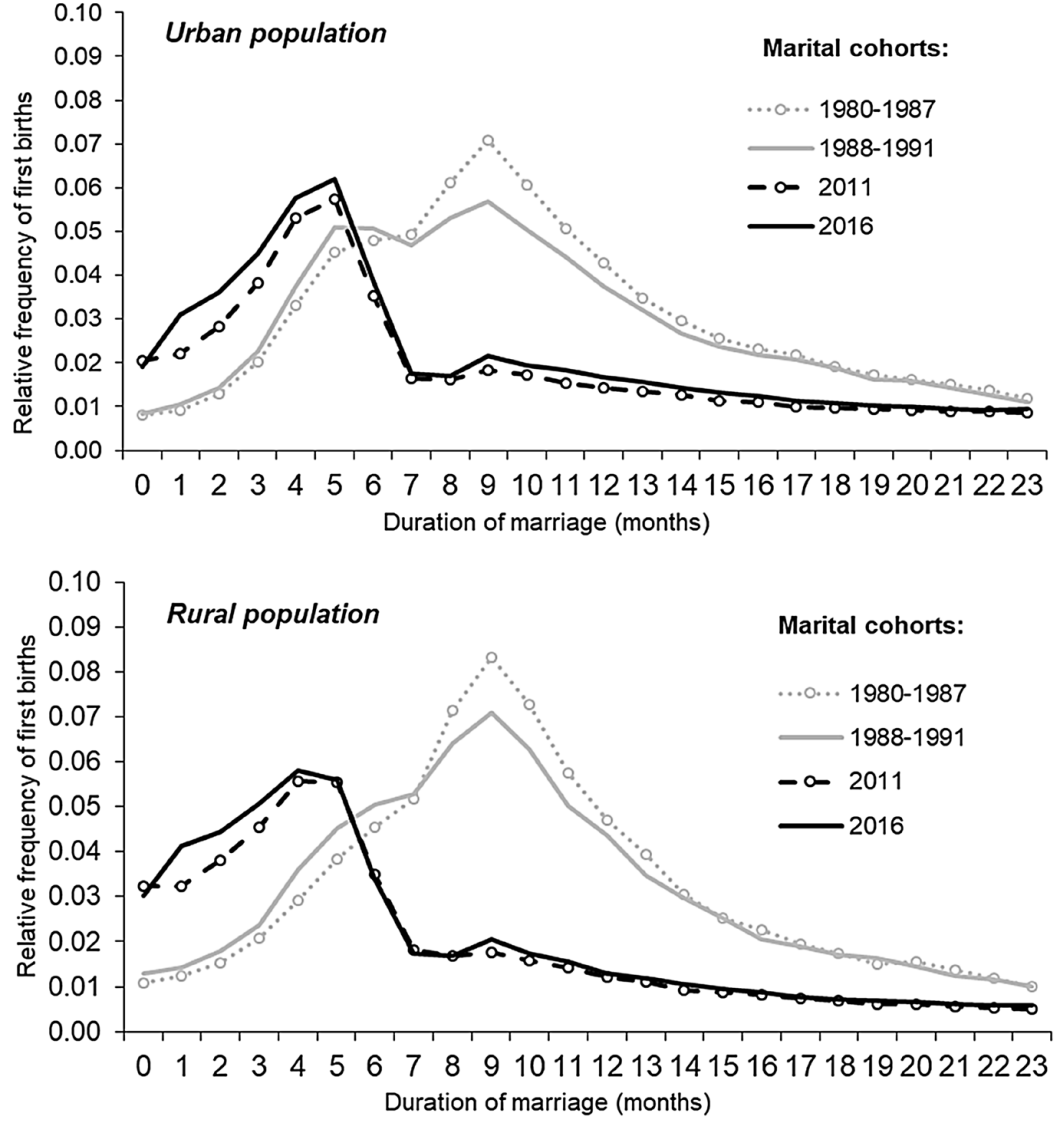
Relative frequency of first births born (within two years of marriage) to women who married at ages under age 35 by duration of marriage and urban–rural place of residence *Data sources:* the 1994 microcensus; data on birth records for 2000–2002, 2011–2013, and 2016–2018; official statistics on the number of marriages by the age of the bride

A comparison of the childbearing patterns in urban and rural areas (Fig. 3) showed that the changes in the pattern of the timing of the first (marital) birth started earlier in urban areas. Among the 1980–1987 marital cohort, the first birth occurred most frequently in the ninth month of marriage in both urban and rural areas, which demonstrates the homogeneity of sexual, reproductive, and marital behaviour in Russia at that time. The shift in the interrelationship between marriage and conception and thus towards an increasing share of conceptions occurring before marriage first became apparent in the urban population of the 1988–1991 marital cohorts. Among this population, almost as many children were born in the fifth month of marriage as in the ninth month of marriage. Among the rural population of the same marital cohorts, the curves remained unimodal, although they were very similar to each other in terms of shape. This leads us to conclude that urban women were substantially ahead of rural women in this shift, as signified by the increasing temporal separation of marriage and conception, and the increasing postponement of marriage in the event of a non-marital pregnancy.

### The Role of Cohabitation

**Fig. 4:**
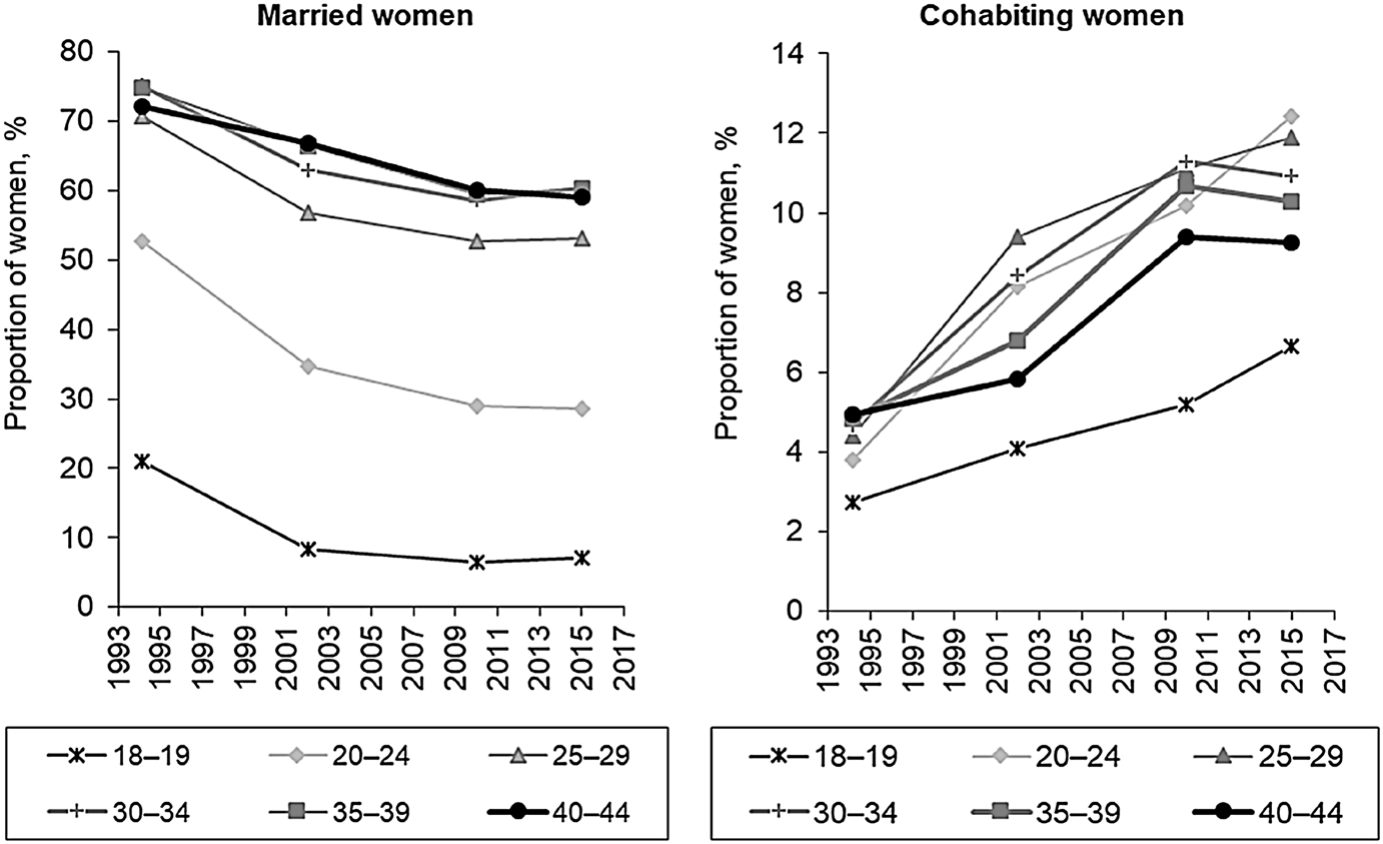
Proportions of married and cohabiting women among all women by age groups. *Data sources*: microcensuses of 1994 and 2015 and censuses of 2002 and 2010

Changes in the timing of the first birth relative to the length of marriage were taking place alongside the shifts in the composition of the female population by marital status. The proportion of women who were married was consistently declining from one population census/microcensus to the other, whereas the proportion of women who were cohabiting was increasing (Fig. 4). The decline in marriage was occurring at a faster pace than the growth in cohabitation. As a result, the total share of partnered women was shrinking, and the share of women in this group who were cohabiting was gradually increasing.

**Fig. 5:**
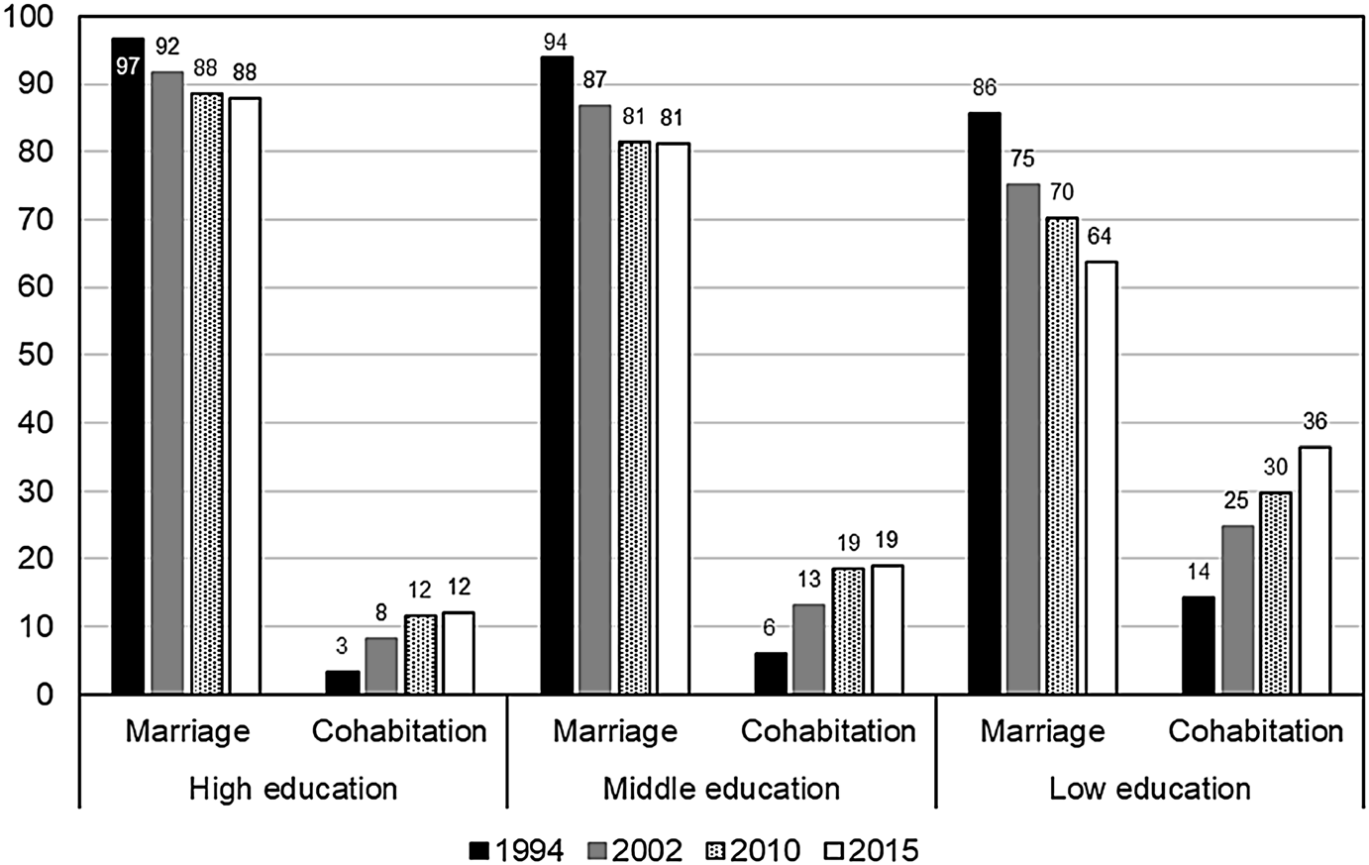
Proportions of women aged 25–34 by attained level of education and partnership status, microcensus and census data (*percent*). *Note*: The high, middle, and low educational categories correspond, respectively, to higher education attained at university or another school of higher education, completed secondary education, and less than secondary education. *Data sources*: microcensuses of 1994 and 2015 and censuses of 2002 and 2010

To shed more light on the question of how advanced the diffusion of cohabitation has become in Russian society, we investigated the variation in the type of partnership by women’s completed education using data from the 1994 and the 2015 microcensuses and the 2002 and the 2010 censuses (Fig. 5). The results of the analysis showed that in 1994, cohabitation was most common among partnered women with low education. Although the share of cohabiting women increased noticeably across all three (high, middle, and low) educational categories from 1994 to 2015, consensual unions were found to be more frequent among women with lower education than among women with higher education over the entire period.

It is important to keep in mind, however, that significant changes have been taking place in the educational structure of the country’s female population, as the share of women with higher education has been increasing in Russia. Among women aged 25–34, the share who were highly educated doubled from the 1994 microcensus to the 2010 census (from 20.8 to 42%, respectively), whereas the share of women in this age group with low education remained relatively stable, at around 4–6%. Thus, it should be emphasised that although the proportion of partnered women who are cohabiting is much higher among those with lower than with higher levels of education, quantitatively, the number of cohabiting women with low education is small.

To sum up, it is apparent that union formation behaviour continues to be socio-economically differentiated in Russia. At the same time, however, cohabitation has been gradually increasing over time, and, as we described above, it has been increasing across all educational groups of women.

**Fig. 6:**
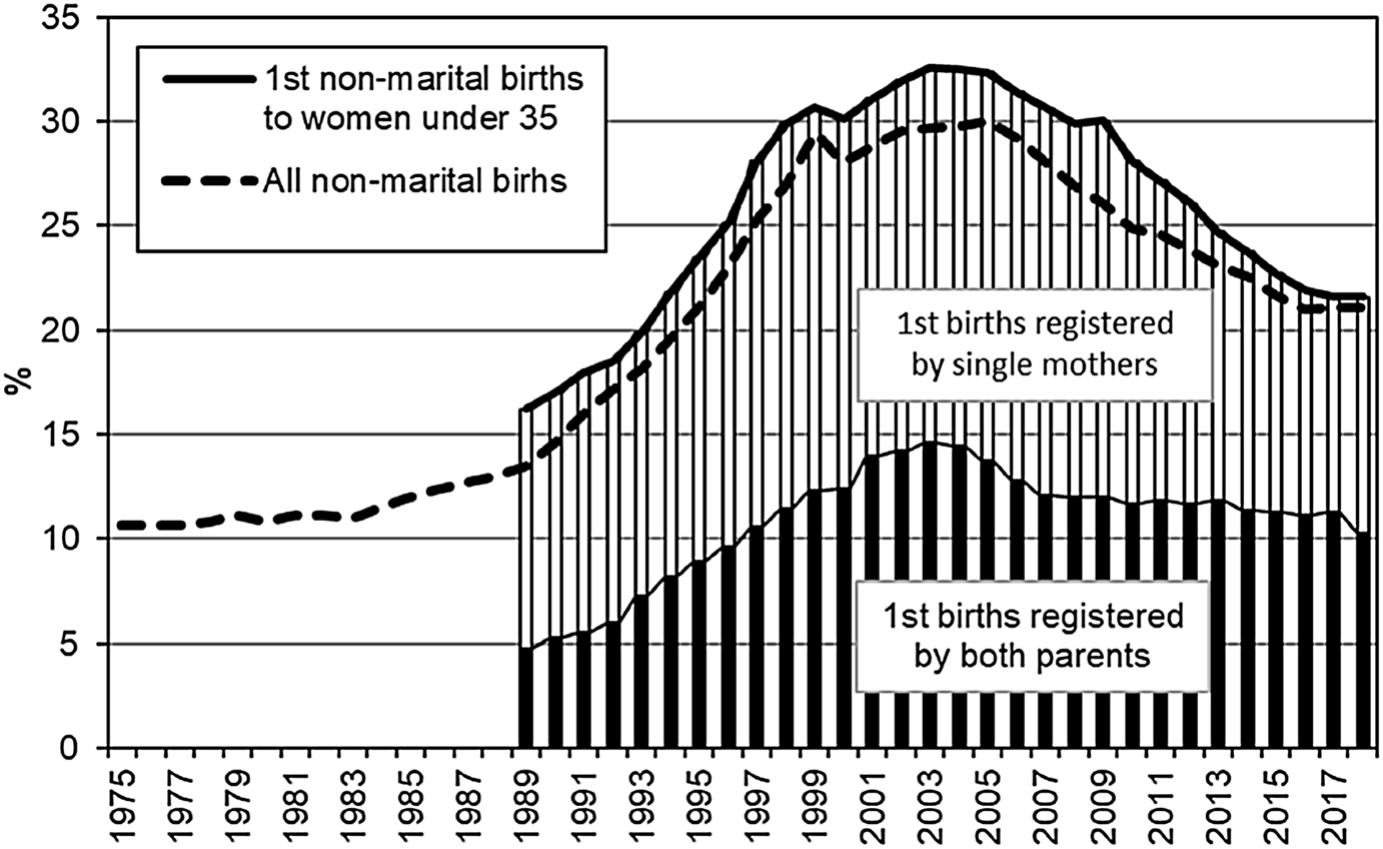
Proportion of non-marital births among all births and among first births to mothers under age 35

As cohabitation has been increasing, non-marital conceptions and non-marital births have been rising as well. As we noted before, the highest non-marital birth level, of 30%, was recorded in 2005 (Fig. 6). When we look at non-marital first births to women aged 35 and younger, we see a similar trend: the share of these births among all first births was increasing throughout the 1990s, was relatively stable for a few years thereafter, and started decreasing after 2009 (Fig. 6).

Figure 6 shows the statistics on non-marital first births by type of birth registration. The changes in the share of first births registered by single mothers (the name of the child’s father is provided based the mother’s statement only, which has no legal consequences, or the child’s father is not identified) closely followed the dynamics for all non-marital births. According to the 2018 statistics, the share of non-marital first births registered by single mothers had declined almost to the 1989 level (see Appendix 3 in the online supplementary materials). The trend in first births registered by both parents looked slightly different. The share of these births was growing until 2002, then stabilised for a few years, and then declined. This period of decline was, however, brief. Since 2007, the trend in non-marital births registered by both parents has been remarkably stable.

## Discussion and Conclusions

Our study provides the most comprehensive empirical evidence (that is available for Russia to date) on changes in premarital conceptions and in first birth intervals among couples who married over a period of almost 60 years, i.e. from 1960 to 2016. For our analysis, we drew on data from multiple accessible sources, including individual-level data from the 1994 Russian microcensus and micro-datasets of birth registration records for select years. Our finding that in Russia, the frequency of first premarital conceptions has not only been increasing, but has surpassed the frequency of marital first conceptions, highlights important shifts in sexual behaviour, as well as in union formation and childbearing in Russia. The turning point in these trends—i.e. the point at which the share of first births that were premaritally conceived surpassed the share of first births that were maritally conceived—was in the mid-1990s, which was also the point in time in Russia when abortion rates started declining, and the use of modern contraception started increasing (Vishnevsky et al., 2017). These contradictory trends suggest that in contemporary Russia, non-marital conceptions (including those resulting in marital births), or at least an increasingly larger share of them, are not unintentional, but are, rather, planned and expected by couples.

Non-marital childbearing in Russia increased steeply (up to 30%) in the 1990–2005 period, and the decrease in the level (to slightly above 20%) that occurred in the following years can largely be explained by the increase in the share of second and higher order births among all births, which rarely happen outside of marriage (Frejka & Zakharov, 2013). The pattern of non-marital childbearing in Russia has undergone significant changes. These changes appear to support our assumption that in today’s Russia, non-marital conceptions are as likely to be planned and expected as conceptions that occur within marriage. Until recently in Russia, more non-marital first births were registered by single mothers than by cohabiting parents. Still in the 2000s, the non-marital childbearing pattern in Russia was characterised by features typical of the “pattern of disadvantage” (Perreli-Harris & Gerber, 2011), i.e. a majority of non-marital first births were to single mothers. However, from about the mid-2010s onwards, this pattern has been changing, i.e. the share of non-marital first births to unmarried partners has been increasing, and the shares of non-marital first births to single mothers and to cohabiting partners have converged. Currently, about one-half of non-marital first births in Russia are to single mothers, while the other half are to unmarried parents. The increasing use of effective contraception in Russia implies that growing numbers of Russian women have reliable means to prevent unwanted pregnancy. Thanks to effective contraception, fewer conceptions are likely to occur in uncommitted relationships or unstable non-marital unions, which typically result in single motherhood. This leads us to the logical inference that most non-marital first births that are jointly registered by unmarried parents were the product of intentional conceptions.

Our finding that among the marital cohorts until about 1987 the peak of the incidence of first births was in the ninth month of marriage points to the relative uniformity of sexual, reproductive, and union formation behaviour in Soviet Russia. The average duration of pregnancy at entry into marriage was three months, which suggests that in the event of a non-marital conception, couples tended to marry shortly after the pregnancy was confirmed by a doctor. This haste indicates that these were “shotgun marriages”, i.e. marriages that were contracted to legitimate the birth of a child, and to protect both the mother and the child from social disapproval.

The 1988–1991 marital cohort can be considered transitional. The first children born to the women of this marital cohort were most likely to be born in the fifth or the ninth month of marriage. It is important to keep in mind that these marriages were contracted in the turbulent years of Perestroika, which were characterised not only by extensive political and economic restructuring, but also by a reduction in censorship and increased levels of freedom of speech. Thus, during this period, Russians gained access to various sources of previously restricted or forbidden information, and they were increasingly able to discuss questions of a private nature, including issues related to family planning, which had previously been considered socially unacceptable. Consensual unions were also becoming more common, and, with the liberalisation of the procedure for registering the place of residence and the development of the housing market, the conditions for creating a joint household improved considerably for unmarried couples. Correspondingly, there was an increase in the share of first births that were premaritally conceived, with the share approaching that of first births conceived within marriage.

The results of our analysis of premarital conceptions among women of the 2000, 2011, and 2016 marital cohorts point to the emergence of a new pattern of conception-induced union formation, which is distinct from the Soviet pattern of shotgun marriages in the conventional sense. While prospective parents in Russia continue to prefer that their child is born within marriage, given the increase in the average duration between conception and marriage, it appears that they are no longer in a hurry to get married. For example, in 2011, the brides who married while pregnant were, on average, in the late fifth month of pregnancy (see Sect. 4.1).

With modern diagnostics, a pregnancy can be now confirmed at a very early stage, and at little expense. Moreover, if the female partner is pregnant, the couple are permitted to marry on the day they file their application if they wish to do so. However, the results of our analysis demonstrate that in such cases, the marriage is often postponed. The tendency to postpone marriage in the event of a pregnancy indicates that pregnancy has ceased to be an unconditional signal for the partners to legitimise their relationship, and that non-marital pregnancy is no longer considered a disgrace that should be hidden from others. This finding is in line with the results of previous research on this topic (Chernova & Shpakovskaya, 2010), which suggest that the connotations of a shotgun marriage have been changing in Russia and that premarital conceptions are increasingly likely to result from rational joint decisions made by long-term cohabitors. Unfortunately, there is little existing research on wedding traditions and practices in Russia. It is possible that the lengthening of the interval between conception and marriage reflects the desire of couples to have more time to plan a proper wedding. However, the results of public opinion polls indicate that the importance of having a wedding ceremony has diminished considerably among young Russians and that couples are often not willing to spend a lot of money on a wedding (VCIOM, 2017).

We found that the interval between conception and entry into marriage started lengthening earlier among the urban population and was more advanced among the urban than the rural population. This gap could be explained by differences in the socio-economic structures of the urban and the rural female populations. The shares of women with higher levels of education, who typically represent the vanguard for changes in demographic behaviour, are considerably higher in urban than in rural areas. Moreover, compared to their rural counterparts, Russian women who live in urban areas have generally had easier access to reproductive health services and information about modern methods of contraception, especially in the 1980–1990s.

The enduring importance in Russia of the link between marriage and childbearing is far from easy to explain. To gain a better understanding of this phenomenon, additional and more in-depth research is needed. It is, however, important to bear in mind that the Russian context differs significantly from that of many other European countries where non-marital partnerships are recognised by law. The socio-economic profile of cohabiting couples has been gradually changing (e.g. the share of cohabitors with higher education has been increasing), which suggests that the diffusion of cohabitation has been progressing in Russian society. However, the lack of legal protections for cohabiting couples remains an important barrier to cohabitation becoming equivalent to a marital union. The Russian Federation does not provide for the legal registration of cohabitation without marriage. This means that the property and financial arrangements, as well as the obligations of and the disputes between cohabiting partners, are not subject to regulation. Consequently, cohabitation is associated with higher levels of insecurity for the partners if one of the partners commits an indiscretion, and for the child(ren) of the partners in the event of a union dissolution. Therefore, in Russia, marriage continues to provide greater stability and security for family members than cohabitation.

Our study is not without limitations, many of which are related to the data we used. First, the birth record datasets we used to examine fertility in the 2000, 2011, and 2016 marital cohorts did not include data on the partnership histories of women. For this reason, we were not able to investigate the risk of premarital conceptions by women’s union status. Moreover, because of a lack of data, we were not able to examine the association between the risk of premarital conception and women’s educational attainment at entry into marriage. We also believe that the phenomenon of premarital conceptions in Moscow, which is a vanguard city by many parameters, deserves a separate investigation. The union formation and childbearing behaviour of women residing in Moscow is likely to differ significantly from that of women living not only in rural areas, but also in other urban areas of Russia. Finally, the limited scope of our study did not allow us to answer the question of why Russian couples prefer to have children within marriage, or of why couples are increasingly postponing marriage in the event of a premarital pregnancy. By using multiple data sources, we were able to cover a long historical period in Russia. Unfortunately, however, the data contain a very limited number of explanatory variables. Nonetheless, we put considerable effort into identifying the main forces that could be responsible for the unique childbearing pattern in Russia and for the continuous preference among expectant parents to marry before their first child is born. We hope that the reasoning and the reflections provided in this study will stimulate and inform further research on this topic.

## Supplementary Information

Below is the link to the electronic supplementary material.

Supplementary file1 (PDF 449 KB)

## Data Availability

The use of data from the 1994 Russian microcensus and data from the 2000, 2011, 2016 birth registration records was authorised for the present study by the Russian Federal State Statistics Service; they are not publicly available. Data from the 2002 and 2010 population censuses and the 2015 microcensus are available freely online.

## Footnotes

1 The Russian Soviet Federative Socialist Republic, called also the Russian SFSR, the RSFSR, or the Soviet Russia, was the largest republic of the Soviet Union. The Russian SFSR was renamed the Russian Federation (or Russia) on 25 December 1991.

2 Komsomol is an abbreviation for the All-Union Leninist Young Communist League, which was a political youth organisation in the Soviet Union.

3 Levada Center (https://www.levada.ru/en/) is a Russian non-governmental research organisation committed to sociological research. It is one of the largest Russian research centres in the field, encompassing a network of 67 regional offices.

4 The Family and Fertility Survey was conducted in 2009, and the survey on the reproductive plans of the Russian population was conducted in 2012 and 2017 by Rosstat.

5 It is important to note here that these shares of planned pregnancies by women’s union status may differ considerably if the women had been asked the question before the pregnancy, because many cohabiting women still choose to marry in the event of a pregnancy, even if it was planned.

6 More information on the 1994 microcensus data can be found in Volkov (1999) and Kharkova and Andreev (2000).

7 We are aware that this definition may include some classification errors, as some maritally conceived births may be premature. According to the official medical statistics, 4.5% of all childbirths in 2011 and 5.8% in 2016 were premature (born at 22–37 weeks of gestation). However, it is noteworthy that there are no statistics in Russia on the survival rates among infants born before 37 weeks of gestation. Moreover, until 2012, all newborns with a body weight of less than 1000 g who died during the first week of life were recorded as stillbirths in Russia.

8 Calculations can be provided upon request.
