# Supplementary material for: Partnership Context of First Births in Russia: The Enduring Significance of Marriage
Source: Eur J Popul. 2021 Dec 10;38(1):37–58. doi: 10.1007/s10680-021-09600-5 (PMC8924331; doi:10.1007/s10680-021-09600-5)
Supplement: Supplementary file 1 — Supplementary file1 (PDF 449 KB) [file 10680_2021_9600_MOESM1_ESM.pdf]

Appendix 1

Table 1 Relative frequency of first births born (within two years of marriage) to women who married at ages under 35 years by marital cohort and duration of marriage.

Year of marriage	Duration of marriage (*months*)											
	0	1	2	3	4	5	6	7	8	9	10	11
1960	0.009	0.006	0.006	0.008	0.009	0.012	0.016	0.023	0.049	0.070	0.064	0.051
1961	0.009	0.007	0.007	0.008	0.010	0.012	0.018	0.027	0.055	0.075	0.066	0.054
1962	0.009	0.006	0.007	0.009	0.010	0.012	0.019	0.029	0.055	0.075	0.064	0.056
1963	0.009	0.006	0.007	0.008	0.011	0.012	0.018	0.029	0.053	0.073	0.064	0.054
1964	0.010	0.007	0.007	0.009	0.011	0.016	0.019	0.029	0.050	0.069	0.062	0.052
1965	0.009	0.007	0.008	0.009	0.010	0.015	0.020	0.029	0.048	0.064	0.059	0.048
1966	0.010	0.008	0.009	0.010	0.015	0.018	0.023	0.032	0.050	0.071	0.063	0.051
1967	0.010	0.008	0.009	0.011	0.012	0.020	0.023	0.034	0.054	0.066	0.059	0.045
1968	0.008	0.007	0.010	0.011	0.015	0.019	0.025	0.033	0.052	0.064	0.058	0.046
1969	0.008	0.007	0.009	0.012	0.016	0.022	0.029	0.036	0.058	0.071	0.062	0.053
1970	0.007	0.007	0.008	0.011	0.014	0.019	0.026	0.033	0.052	0.064	0.057	0.048
1971	0.008	0.007	0.009	0.012	0.017	0.023	0.031	0.039	0.061	0.076	0.064	0.053
1972	0.008	0.008	0.011	0.012	0.019	0.025	0.030	0.039	0.057	0.070	0.060	0.051
1973	0.007	0.008	0.010	0.012	0.019	0.027	0.034	0.040	0.060	0.077	0.066	0.052
1974	0.007	0.007	0.009	0.012	0.020	0.026	0.032	0.039	0.057	0.071	0.061	0.050
1975	0.006	0.007	0.009	0.013	0.020	0.028	0.033	0.039	0.057	0.071	0.064	0.053
1976	0.008	0.008	0.010	0.016	0.022	0.030	0.035	0.040	0.058	0.071	0.062	0.052
1977	0.008	0.008	0.011	0.016	0.023	0.033	0.040	0.041	0.063	0.076	0.065	0.056
1978	0.008	0.010	0.011	0.016	0.024	0.033	0.037	0.043	0.057	0.068	0.063	0.051
1979	0.008	0.009	0.011	0.016	0.026	0.035	0.040	0.044	0.059	0.070	0.061	0.051
1980	0.008	0.008	0.011	0.017	0.024	0.031	0.034	0.037	0.051	0.061	0.053	0.043
1981	0.009	0.009	0.012	0.017	0.027	0.035	0.039	0.043	0.058	0.064	0.057	0.050
1982	0.008	0.010	0.013	0.018	0.026	0.036	0.039	0.042	0.055	0.064	0.056	0.046
1983	0.008	0.010	0.013	0.018	0.028	0.036	0.041	0.041	0.053	0.065	0.054	0.045
1984	0.009	0.010	0.014	0.018	0.028	0.038	0.040	0.042	0.053	0.058	0.053	0.043
1985	0.009	0.009	0.011	0.019	0.029	0.038	0.038	0.039	0.050	0.057	0.049	0.041
1986	0.008	0.011	0.014	0.020	0.028	0.037	0.040	0.040	0.048	0.055	0.046	0.039
1987	0.009	0.010	0.013	0.020	0.030	0.038	0.040	0.041	0.050	0.055	0.049	0.040
1988	0.009	0.011	0.015	0.020	0.031	0.040	0.042	0.040	0.048	0.053	0.046	0.040
1989	0.009	0.011	0.015	0.021	0.033	0.042	0.043	0.040	0.046	0.051	0.045	0.037
1990	0.009	0.011	0.014	0.021	0.032	0.039	0.038	0.037	0.042	0.045	0.041	0.035
1991	0.010	0.011	0.014	0.021	0.033	0.042	0.039	0.036	0.041	0.044	0.042	0.037
2000	0.023	0.017	0.021	0.031	0.043	0.048	0.035	0.021	0.018	0.019	0.017	0.015
2011	0.024	0.025	0.031	0.040	0.054	0.057	0.035	0.017	0.016	0.018	0.017	0.015
2016	0.022	0.033	0.038	0.046	0.058	0.061	0.038	0.017	0.017	0.021	0.019	0.018

Table 1 (*continued*) Relative frequency of first births born (within two years of marriage) to women who married at ages under 35 years by marital cohort and duration of marriage.

Year of marriage	Duration of marriage (*months*)											
	12	13	14	15	16	17	18	19	20	21	22	23
1960	0.042	0.033	0.028	0.024	0.022	0.019	0.018	0.018	0.020	0.020	0.018	0.016
1961	0.044	0.034	0.029	0.025	0.020	0.019	0.018	0.018	0.021	0.020	0.017	0.015
1962	0.044	0.034	0.031	0.024	0.023	0.020	0.019	0.018	0.018	0.018	0.015	0.015
1963	0.046	0.035	0.029	0.024	0.023	0.019	0.019	0.017	0.018	0.018	0.015	0.013
1964	0.041	0.034	0.029	0.024	0.021	0.019	0.018	0.017	0.017	0.018	0.014	0.012
1965	0.040	0.033	0.027	0.024	0.021	0.019	0.018	0.019	0.018	0.018	0.015	0.012
1966	0.042	0.035	0.029	0.025	0.023	0.020	0.019	0.018	0.017	0.018	0.014	0.012
1967	0.042	0.035	0.028	0.026	0.024	0.021	0.018	0.019	0.019	0.017	0.015	0.012
1968	0.040	0.033	0.027	0.025	0.022	0.021	0.020	0.019	0.019	0.017	0.016	0.012
1969	0.044	0.034	0.031	0.026	0.023	0.020	0.019	0.018	0.016	0.016	0.014	0.011
1970	0.039	0.032	0.026	0.023	0.022	0.021	0.020	0.019	0.019	0.020	0.017	0.015
1971	0.045	0.035	0.029	0.025	0.022	0.020	0.020	0.018	0.017	0.016	0.015	0.012
1972	0.042	0.033	0.029	0.024	0.021	0.020	0.019	0.018	0.018	0.017	0.014	0.013
1973	0.040	0.034	0.030	0.024	0.022	0.019	0.018	0.017	0.017	0.015	0.013	0.012
1974	0.044	0.036	0.029	0.026	0.023	0.020	0.019	0.017	0.016	0.015	0.014	0.013
1975	0.044	0.037	0.030	0.026	0.023	0.022	0.020	0.017	0.017	0.016	0.013	0.012
1976	0.043	0.034	0.029	0.025	0.023	0.021	0.020	0.018	0.017	0.016	0.014	0.012
1977	0.044	0.036	0.030	0.026	0.023	0.021	0.020	0.017	0.016	0.015	0.012	0.011
1978	0.042	0.034	0.028	0.025	0.022	0.020	0.018	0.017	0.016	0.014	0.013	0.011
1979	0.043	0.034	0.029	0.024	0.022	0.020	0.017	0.016	0.014	0.013	0.012	0.010
1980	0.036	0.031	0.026	0.022	0.021	0.021	0.017	0.016	0.017	0.015	0.014	0.013
1981	0.039	0.032	0.027	0.023	0.021	0.019	0.015	0.014	0.014	0.014	0.012	0.010
1982	0.038	0.031	0.027	0.023	0.021	0.018	0.018	0.015	0.014	0.013	0.012	0.010
1983	0.038	0.031	0.026	0.022	0.020	0.017	0.016	0.014	0.014	0.013	0.012	0.010
1984	0.038	0.031	0.025	0.022	0.018	0.017	0.016	0.014	0.013	0.011	0.010	0.009
1985	0.036	0.031	0.024	0.022	0.018	0.018	0.016	0.013	0.013	0.012	0.010	0.010
1986	0.036	0.028	0.023	0.020	0.018	0.017	0.015	0.014	0.013	0.012	0.010	0.009
1987	0.034	0.028	0.024	0.021	0.019	0.018	0.016	0.013	0.014	0.013	0.011	0.009
1988	0.036	0.029	0.025	0.022	0.020	0.018	0.016	0.014	0.013	0.011	0.010	0.009
1989	0.031	0.027	0.024	0.021	0.018	0.017	0.015	0.013	0.013	0.011	0.010	0.009
1990	0.031	0.025	0.022	0.019	0.019	0.018	0.016	0.014	0.014	0.014	0.011	0.010
1991	0.031	0.027	0.020	0.020	0.017	0.017	0.015	0.013	0.013	0.012	0.011	0.008
2000	0.013	0.012	0.011	0.010	0.009	0.008	0.007	0.007	0.007	0.006	0.006	0.006
2011	0.014	0.013	0.012	0.011	0.010	0.009	0.009	0.009	0.008	0.008	0.008	0.008
2016	0.016	0.015	0.014	0.012	0.012	0.010	0.010	0.009	0.009	0.009	0.008	0.009

Appendix 2

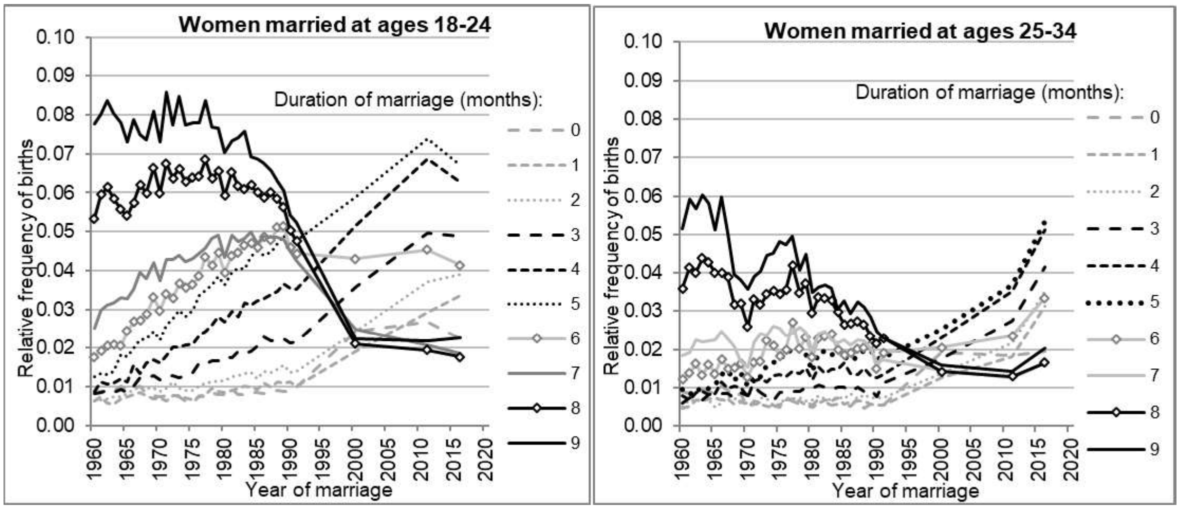

Fig. 1 Relative frequency of first births born (within two years of marriage) to women who married at ages 18-24 and 25-34 by marital cohort and duration of marriage (up to ninth month)

Appendix 3

Table 2 Proportion of non-marital births among first births (*percent*)

	Mothers of all ages			Mother under 35		
	All births	Registered by both parents	Registered by single mother	All births	Registered by both parents	Registered by single mother
1989	16.7	4.8	11.9	16.2	4.8	11.4
1990	17.5	5.3	12.1	17.0	5.3	11.7
1991	18.4	5.5	12.9	18.0	5.5	12.4
1992	18.9	6.0	12.9	18.5	6.0	12.5
1993	20.3	7.3	13.0	19.9	7.3	12.6
1994	22.1	8.1	13.9	21.8	8.2	13.6
1995	23.8	8.9	14.9	23.5	8.9	14.6
1996	25.5	9.6	15.9	25.1	9.7	15.5
1997	28.3	10.6	17.7	28.1	10.6	17.5
1998	30.1	11.5	18.6	29.9	11.5	18.4
1999	31.1	12.4	18.7	30.7	12.3	18.3
2000	30.5	12.5	18.0	30.1	12.4	17.7
2001	31.5	14.1	17.4	31.1	14.0	17.1
2002	32.3	14.3	18.0	32.0	14.2	17.8
2003	32.9	14.8	18.1	32.6	14.6	17.9
2004	32.8	14.5	18.3	32.5	14.4	18.0
2005	32.7	13.9	18.8	32.4	13.8	18.6
2006	31.8	12.9	18.9	31.4	12.8	18.7
2007	31.0	12.2	18.8	30.7	12.1	18.6
2008	30.3	12.1	18.2	29.9	12.0	17.9
2009	30.4	12.1	18.3	30.0	12.0	18.0
2010	28.6	11.9	16.8	28.1	11.7	16.4
2011	27.7	12.0	15.7	27.2	11.8	15.4
2012	26.7	11.9	14.7	26.1	11.7	14.4
2013	25.4	12.2	13.2	24.7	11.9	12.8
2014	24.5	11.7	12.8	23.7	11.4	12.3
2015	23.6	11.6	11.9	22.7	11.3	11.5
2016	22.9	11.6	11.3	21.9	11.1	10.8
2017	22.6	11.8	10.8	21.6	11.3	10.3
2018	22.7	10.8	11.9	21.6	10.3	11.3

Source: own calculations based on data from Demographic Yearbook of Russia (2019)
